# Evolution Model of Health Food Safety Risk Based on Prospect Theory

**DOI:** 10.1155/2018/8769563

**Published:** 2018-10-23

**Authors:** Jun Luo, Baichao Ma, Yongle Zhao, Tingqiang Chen

**Affiliations:** ^1^School of Health Economics and Management, Nanjing University of Chinese Medicine, Nanjing, China; ^2^Business School, Hohai University, Nanjing, China; ^3^School of Business, Nankai University, Tianjin, China; ^4^School of Economics and Management, Nanjing Tech University, Nanjing, China

## Abstract

In the growing market of health food, certain disturbances occur, such as uneven quality of products, imitation of health food, prohibited drug content in health food, functional efficacy, and actual disagreement. The safety of health food has attracted wide attention from all walks of life. In this study, we constructed a three-party game model of health food safety risk evolution, which includes health food enterprises, health food consumers, and government regulators, based on prospect theory and evolutionary game method. We also consider the attributes of “trust products” of health food, the ability to identify the safety information of health food, the subjective perception of the efficacy of health food, and the certification effect of the regulatory information of the government supervision department. The influence mechanism of these factors, including the cost of searching for health food information, consumers' subjective perception of health food efficiency, and the certification effect of supervision departments, on health food safety risk evolution is described using theoretical deduction and simulation analysis. On this basis, the corresponding conclusions are established, which provide a theoretical basis for further exploration of the strategy of health food market governance.

## 1. Introduction

The occurrence of aging society and the current health needs of people have led to the improvement of the health food market and the promotion of the development of the health food industry in China. However, certain disturbances have arisen in the process of the growing market of health food, such as uneven quality of products, imitation of health food, prohibited drug content in health food, functional efficacy, and actual disagreement. The safety of health food has attracted widespread attention from all walks of life. The quality and safety of health food in the health food market are direct threats to the life and safety of people. At the same time, the healthy development of the economy and the harmony and stability of the society are seriously restricted. In July 2017, the State Council Food Safety Office and nine other departments conducted a food and health fraud and false propaganda and rectification actions in the entire country. Thus far, the regulatory authorities have handled and published over 8000 administrative penalty cases.

The illegal behavior of health food enterprises is a direct factor of health food safety risks and is mainly driven by excess interests. Making a timely and accurate judgment on the factual quality of health food for regulators and consumers is difficult because of the credence and experience goods attributes of health food [1, 2]. The probability in which the illegal behavior of health food enterprises has been recognized is relatively small, which results in a serious adverse selection problem in the health food market. Meanwhile, the willingness of food consumers to pay is often low because of the difficulty in identifying the quality of health food. In addition, the cost of health food enterprises for the production of high-quality health food is difficult to establish or even be expelled by low-quality health food [3] and results in the situation of using “bad money” to expel “good money” [4] Third, the existence of information asymmetry in the health food market provides a way for the production and diffusion of health food safety risk, which is also the main reason for the frequent occurrence of health food safety problems [5, 6]. The problem of information asymmetry in the health food market is not only between upstream and downstream health food enterprises but also between health food enterprises and government supervision departments. This problem also exists between health food enterprises and its consumers, which is the most severe [7, 8]. In addition, making accurate judgments of food quality for consumers is difficult because of the credence good attribute of health food.

In view of the credence good attribute of health food, several scholars have proposed that the government should increase the intensity of administrative and judicial punishment for the protection of health food safety [9], establish all types of international certification system [10, 11], and improve the transparency of health food information [12]; [13]. At the same time, the mutual coupling of numerous factors, including information opacity and weak social supervision, leads to the difficulty in attaining satisfactory supervision results of the health food market. In addition, integrating all social forces is necessary for health food safety management [14, 15]. Only by giving full play to all social forces can the consumers obtain sufficient information to determine the quality of health food in the health food market, and government supervision departments are further targeted to conduct regulatory activities [16, 17].

Therefore, we construct a strategic game model, in which the parties include government regulators, health food consumers, and health food enterprises, combined with the credence good attribute of health food, the problem of serious information asymmetry in the health food market, and the special attributes of health food. Factors, such as determining consumers' ability to identify health food safety information, the subjective perception of the efficacy of health food, and the certification effect of regulatory information in the government supervision department, are also considered. We depict the contagion mechanism of health food safety risk using the proposed game model and provide the theoretical support for further discussion of the corresponding strategies for ensuring the safety of health food.

The remainder of this paper is organized as follows.  Section 2 establishes the corresponding model assumptions and relevant economic analysis and combines the prospect theory and the actual situation of the health food market. In Section 3, a three-party evolutionary game model, which includes health food enterprises, health food consumers, and government supervision departments, is initially constructed. On this basis, the influence factors of health food safety and the evolution mechanism of health insurance are analyzed via mathematical analysis and simulation experiments.  Section 4 summarizes the conclusions of this study.

## 2. Model Assumptions

We model the health food consumption market into an evolutionary game model, which includes the government, health food enterprises, and consumers, without considering the influence of other external factors. In our game model, the explicit participants are the consumers and health food enterprises. At the same time, the government supervision department can exert a certain level of influence on consumers' consumption behavior and the income of health food enterprises through the publication of relevant information about such enterprises. Therefore, the government supervision department is an implicit participant in this model.

Health food cannot improve the physical fitness of people in a short time, such that prolonging life and regulating the function of the human body takes a long time. Thus, consumers' judgment on the efficacy of health food will be influenced by their own subjective perception, which also directly affects the behavior of health food consumers [18, 19]. Prospect theory has strong applicability in describing and predicting behaviors that are inconsistent with traditional expectation and expected utility theories in decision-making [20, 21]. Therefore, using prospect theory can reasonably depict the utility function of the behavior of health food consumers. On the basis of the aforementioned considerations, the basic assumptions of this paper are as follows.

**Assumption 1 .**

Health food consumers make consumption decisions based on the value function of prospect theory.

Health food cannot improve the physique of people in a short time, such that prolonging life and regulating the function of the human body can take a long time. The prospect value function of health food for consumers can be expressed as *V*=∑_*i*_*π*(*p*_*i*_)*v*(Δ*ω*_*i*_), where *p*_*i*_ is the probability of health food safety and efficiency, *π*(*p*_*i*_) is the subjective cognition of health food, and *v*(Δ*ω*_*i*_) is the value function of the consumer health food prospect. In addition, *e* denotes the effects of consuming qualified health food, which is the cost of the need to pay in addition to the price of health food, and *c* represents the cost of searching information. If consumers buy low-quality health food, then they will not only lose health benefits but will also incur other losses; *L* denotes such losses. If consumers do not buy health food and need to pay other costs to achieve longevity, then we assume these costs *R* as consumer losses.

**Assumption 2 .**

Regulatory authorities can play a certification role by issuing relevant information on health food enterprises.

For health food enterprises with good faith, releasing relevant information about them through the government supervision department has a certain degree of certification effect, which affects consumer behavior. In the decision-making of consumers, consumers will increase their purchasing power on products of good faith and health food enterprises and then improve the benefits of good faith management of health food enterprises. We assume that the coefficient of the certification effect is *δ*(*δ* ≥ 1). For health food enterprises with poor reputation, we assume that the coefficient of the certification effect is *λ*(*λ* < 1).

**Assumption 3 .**

Health food enterprises determine the production units of health food according to the demand function.

*P*_h_ denotes the price of high-quality health food, and the price of low-quality health food is expressed as *P*_l_(*P*_h_ > *P*_l_); under these two selling prices, the demand function of health food is *Q*_h_=*a* − *bP*_h_ and *Q*_h_=*a* − *bP*_l_, respectively. The production costs of high- and low-quality health food are *C*_h_ and *C*_l_, respectively.

## 3. Model and Analysis

### 3.1. Model

High-quality health food holds a health effect, whereas low-quality health food not only has no health effect but its quality also holds problems and will directly harm consumers' safety.

From Table 1, we can get

(1) $$\begin{array}{c} V_{1}\left(p_{1},e,c\right)=\pi \left(p_{1}\right)v_{1}\left(e-c\right)+\pi \left(1-p_{1}\right)v_{1}\left(0\right)=\pi \left(p_{1}\right)v_{1}\left(e-c\right),\\ V_{2}\left(p_{2},L,c\right)=\pi \left(p_{2}\right)v_{2}\left(-L-c\right)+\pi \left(1-p_{2}\right)v_{2}\left(0\right)=\pi \left(p_{2}\right)v_{2}\left(-L-c\right). \end{array}$$

### 3.2. Model Analysis

#### 3.2.1. Value Function of Consumers

The value function of consumers who buy health food can be expressed based on the payment matrix of the model as follows:

(2) $$\begin{array}{c} U_{\text{B}1}=y\left[\alpha \pi \left(p_{1}\right)\left(e-c\right)-P_{\text{h}}\right]+\left(1-y\right)\left[\beta \pi \left(p_{2}\right)\left(-L-c\right)-P_{\text{l}}\right]. \end{array}$$

At the same time, the value function of consumers who buy health food can be expressed as follows:

(3) $$\begin{array}{c} U_{\text{B}2}=y\left(-R\right)+\left(1-y\right)\left(-R\right)=-R. \end{array}$$

The mean expected value of consumers can be expressed as

(4) $$\begin{array}{c} \bar{U_{\text{B}}}=x\cdot U_{\text{B}1}+\left(1-x\right)U_{\text{B}2}. \end{array}$$

#### 3.2.2. Value Function of Health Food Enterprises

We can obtain the utility function of health food enterprises that produce high-quality items based on the payment matrix of the model as follows:

(5) $$\begin{array}{c} U_{\text{E}1}=x\delta \left(a-bP_{\text{h}}-C_{\text{h}}\right)P_{\text{h}}-\left(1-x\right)\left(a-bP_{\text{h}}\right)C_{\text{h}}. \end{array}$$

The utility function of health food enterprises that produce low-quality items can be expressed as follows:

(6) $$\begin{array}{c} U_{\text{E}2}=x\lambda \left(a-bP_{\text{l}}-C_{\text{l}}\right)P_{\text{l}}-\left(1-x\right)\left(a-bP_{\text{l}}\right)C_{\text{l}}. \end{array}$$

Then, the mean utility function of health food enterprises can be written as

(7) $$\begin{array}{c} \bar{U_{\text{E}}}=yU_{\text{E}1}+\left(1-y\right)U_{\text{E}2}. \end{array}$$

#### 3.2.3. Equilibrium Analysis of the Dynamic Evolution System

According to the value function of consumers and the utility function of health food enterprises, we can obtain the replicating dynamic equations of the model as follows:

(8) $$\begin{array}{c} \frac{dx}{dt}=x\left(1-x\right)\left(U_{\text{B}1}-U_{\text{B}2}\right), \end{array}$$

(9) $$\begin{array}{c} \frac{dy}{dt}=y\left(1-y\right)\left(U_{\text{E}1}-U_{\text{E}2}\right). \end{array}$$

Then, according to the replicating dynamic equations of the model, the equilibrium points of the system are (0,0), (0,1), (1,0), (1,1), and (*x*^*∗*^, *y*^*∗*^), where the concrete forms of *x*^*∗*^ and *y*^*∗*^ can be expressed as follows:

(10) $$\begin{array}{c} x^{*}=\frac{\left(a-bP_{\text{h}}\right)C_{\text{h}}-\left(a-bP_{\text{l}}\right)C_{\text{l}}}{\delta \left(a-bP_{\text{h}}-C_{\text{h}}\right)P_{\text{h}}+\left(a-bP_{\text{h}}\right)C_{\text{h}}-\lambda \left(a-bP_{\text{l}}-C_{\text{l}}\right)P_{\text{l}}-\left(a-bP_{\text{l}}\right)C_{\text{l}}},\\ y^{*}=\frac{\beta \pi \left(p_{2}\right)\left(-L-c\right)-P_{\text{l}}+R}{\alpha \pi \left(p_{1}\right)\left(e-c\right)-P_{\text{h}}-\beta \pi \left(p_{2}\right)\left(-L-c\right)+P_{\text{l}}}. \end{array}$$

#### 3.2.4. Stability Analysis of the Dynamic Evolution System

The local stability of five equilibrium points is analyzed based on the equilibrium analysis of the model and determined using a Jacobian matrix with the aid of the Friedman method. The Jacobian matrix can be expressed based on Equations (8) and (9) as follows:

(11) $$\begin{array}{c} J=\left[\begin{array}{cc} \frac{\partial \dot{x}}{\partial x} & \frac{\partial \dot{x}}{\partial y}\\ \frac{\partial \dot{y}}{\partial x} & \frac{\partial \dot{y}}{\partial y} \end{array}\right]=\left[\begin{array}{cc} a_{11} & a_{12}\\ a_{21} & a_{22} \end{array}\right], \end{array}$$

where the values of each element in the Jacobian matrix can be expressed as follows:

(12) $$\begin{array}{c} a_{11}=\left(1-2x\right)\left\{y\left[\alpha \pi \left(p_{1}\right)\left(e-c\right)-P_{\text{h}}\right]+\left(1-y\right)\left[\beta \pi \left(p_{2}\right)\left(-L-c\right)-P_{\text{l}}\right]+R\right\},\\ a_{12}=x\left(1-x\right)\left[\alpha \pi \left(p_{1}\right)\left(e-c\right)-P_{\text{h}}-\beta \pi \left(p_{2}\right)\left(-L-c\right)+P_{\text{l}}\right],\\ a_{21}=y\left(1-y\right)\left[\delta \left(a-bP_{\text{h}}-C_{\text{h}}\right)P_{\text{h}}+\left(a-bP_{\text{h}}\right)C_{\text{h}}-\lambda \left(a-bP_{\text{l}}-C_{\text{l}}\right)P_{\text{l}}-\left(a-bP_{\text{l}}\right)C_{\text{l}}\right],\\ a_{22}=\left(1-2y\right)\left[x\delta \left(a-bP_{\text{h}}-C_{\text{h}}\right)P_{\text{h}}-\left(1-x\right)\left(a-bP_{\text{h}}\right)C_{\text{h}}-x\lambda \left(a-bP_{\text{l}}-C_{\text{l}}\right)P_{\text{l}}+\left(1-x\right)\left(a-bP_{\text{l}}\right)C_{\text{l}}\right]. \end{array}$$

From Table 2, we can get

(13) $$\begin{array}{c} \Lambda =\frac{\left[\delta \left(a-bP_{\text{h}}-C_{\text{h}}\right)P_{\text{h}}-\lambda \left(a-bP_{\text{l}}-C_{\text{l}}\right)P_{\text{l}}\right]\times \left[\left(a-bP_{\text{h}}\right)C_{\text{h}}-\left(a-bP_{\text{l}}\right)C_{\text{l}}\right]}{\delta \left(a-bP_{\text{h}}-C_{\text{h}}\right)P_{\text{h}}+\left(a-bP_{\text{h}}\right)C_{\text{h}}-\lambda \left(a-bP_{\text{l}}-C_{\text{l}}\right)P_{\text{l}}-\left(a-bP_{\text{l}}\right)C_{\text{l}}}\times \;\frac{\alpha \pi \left(p_{1}\right)\left(e-c\right)-P_{\text{h}}-\beta \pi \left(p_{2}\right)\left(-L-c\right)+P_{\text{l}}}{\delta \left(a-bP_{\text{h}}-C_{\text{h}}\right)P_{\text{h}}+\left(a-bP_{\text{h}}\right)C_{\text{h}}-\lambda \left(a-bP_{\text{l}}-C_{\text{l}}\right)P_{\text{l}}-\left(a-bP_{\text{l}}\right)C_{\text{l}}},\\ \Gamma =\frac{\left[\alpha \pi \left(p_{1}\right)\left(e-c\right)-P_{\text{h}}-2\beta \pi \left(p_{2}\right)\left(-L-c\right)+2P_{\text{l}}-R\right]\times \left[\beta \pi \left(p_{2}\right)\left(-L-c\right)-P_{\text{l}}+R\right]}{\alpha \pi \left(p_{1}\right)\left(e-c\right)-P_{\text{h}}-\beta \pi \left(p_{2}\right)\left(-L-c\right)+P_{\text{l}}}\times \;\frac{\delta \left(a-bP_{\text{h}}-C_{\text{h}}\right)P_{\text{h}}+\left(a-bP_{\text{h}}\right)C_{\text{h}}-\lambda \left(a-bP_{\text{l}}-C_{\text{l}}\right)P_{\text{l}}-\left(a-bP_{\text{l}}\right)C_{\text{l}}}{\alpha \pi \left(p_{1}\right)\left(e-c\right)-P_{\text{h}}-\beta \pi \left(p_{2}\right)\left(-L-c\right)+P_{\text{l}}}. \end{array}$$

When assessing the symbols of each element in the Jacobian matrix, the judgment is based on the relevant assumptions in the model and the actual health food market, where the main relationships are (1) *απ*(*p*_1_)(*e* − *c*) − *P*_h_+*R* > 0, which indicates that when consumers buy high-quality health food, their benefits will be greater than those who opt not to buy them and (2) *βπ*(*p*_2_)(−*L* − *c*) − *P*_l_+*R* < 0, which implies that when consumers buy low-quality health food, their benefits will be less than those who opt not to buy them. From these two relationships, we can make a systematic judgment and analysis of the stability of the model according to different situations.

**Case 1 .**

(*a* − *bP*_h_)*C*_h_ − (*a* − *bP*_l_)*C*_l_ > 0  and  *δ*(*a* − *bP*_h_ − *C*_h_)*P*_h_ − *λ*(*a* − *bP*_l_ − *C*_l_)*P*_l_ > 0.

The constraint conditions of Case 1 indicate that (1) when consumers choose to buy health food, the enterprises that produce high-quality health food gain increased profit and (2) when consumers opt not to buy health food, the enterprises that produce low-quality health food gain increased profit. Under these conditions, Table 3 presents the results of local stability analysis.

The following conclusions can be drawn from the results of the stability analysis in Table 3.

**Theorem 1 .**

Under the constraint conditions (*a* − *bP*_h_)*C*_h_ − (*a* − *bP*_l_)*C*_l_ > 0 and *δ*(*a* − *bP*_h_ − *C*_h_)*P*_h_ − *λ*(*a* − *bP*_l_ − *C*_l_)*P*_l_ > 0, the stable strategies are ESS (0,0) and ESS (1,1), respectively.

**Figure 1:**
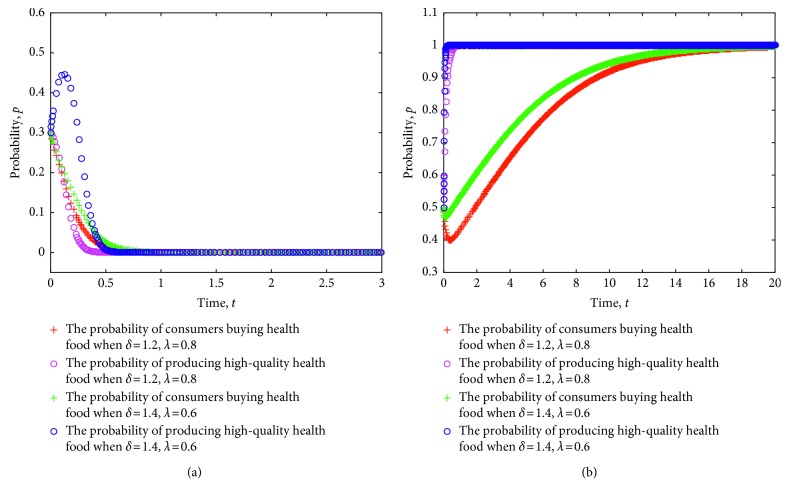
Evolution of strategy of consumers and health food enterprises under different certification effects.

When the evolution system satisfies the constraint conditions in Case 1, this system is locally stable. To verify this notion, we simulate the evolution of the strategy of health food enterprises and consumers using MATLAB software and explore the evolution mechanism of the dynamic system. First, in view of the different certification effects of the relevant information on health food enterprises as issued by the government, the game strategy of consumers and health food enterprises is simulated. The evolution of the strategies of consumers and health food enterprises is obtained, as shown in Figure 1, where the value of each parameter is set as follows: *e*=8, *c*=1, *π*(*p*_1_)=0.9, *π*(*p*_2_)=0.6, *L*=5, *P*_h_=9, *P*_l_=7, *R*=3, *a*=30, *b*=0.3, *C*_h_=5, and *C*_l_=3.5.

Figure 1(a) shows the process in which consumers and health food enterprises are stable in ESS (0,0) when the certification effect varies. From the figure, a stronger certification effect implies slower strategy of health food enterprises in stabilizing the production of low-quality health food. Likewise, a weaker certification effect indicates a faster strategy of health food enterprises in stabilizing the production of low-quality health food. Moreover, a stronger certification effect indicates more profit for companies that produce high-quality health food when consumers continue to consume health food. Once the probability of consumers' purchase of health food in the market is reduced, the effect of certification will be difficult to play. Consequently, health food enterprises will have difficulty in obtaining high income through certification effect and will gradually gain excess returns by reducing production quality and production and operating costs. The strategy will gradually stabilize to produce low-quality health food. In this process, a strong certification effect will delay the strategy of health food enterprises to stabilize low-quality health food. A weaker certification effect indicates the high probability of health food safety risks (Figure 1(a)).

Figure 1(b) shows the process in which consumers and health food enterprises are stable in ESS (1,1) when the certification effect varies. From the figure, a stronger certification effect implies a faster strategy of health food enterprises in producing high-quality health food. Similarly, a weaker certification effect indicates slower strategy of health food enterprises in stabilizing the production of low-quality health food. Moreover, health food enterprises can gain increased benefits through good faith operation because of the strong certification effect in the case of continued consumer purchase of health food, thereby speeding up the strategy of health food enterprises in stabilizing the production of high-quality health food. Conversely, the relatively weak certification effect will delay the speed of the strategy of health food enterprises to be stable. Particularly, a weak certification effect will lead to health food safety risks.

**Figure 2:**
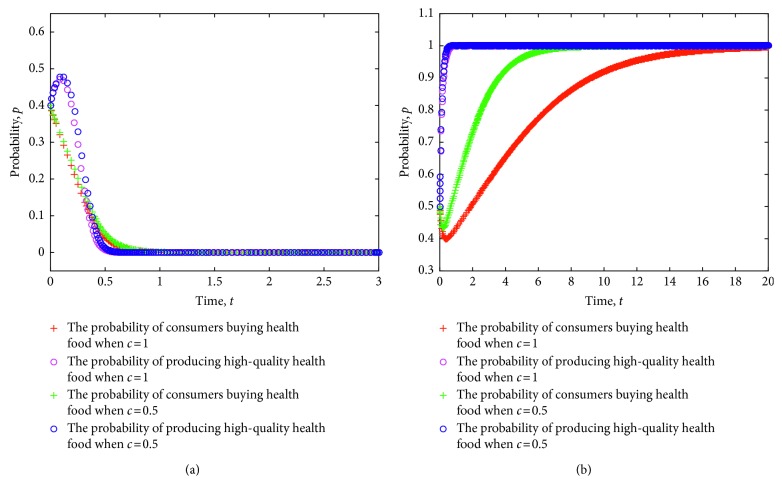
Evolution of strategies of consumers and health food enterprises under different costs of information acquisition.

At the same time, a simulation experiment on the strategy evolution of consumers and health food enterprises is conducted considering the different cost of information acquisition.  Figure 2 shows the simulation results, where the value of each parameter is set as follows: *e*=8, *π*(*p*_1_)=0.9, *π*(*p*_2_)=0.6, *L*=5, *P*_h_=9, *P*_l_=7, *R*=3, *a*=30, *b*=0.3, *C*_h_=5, *C*_l_=3.5, *δ*=1.2, and *λ*=0.8.

Figure 2 shows the evolution of strategies for consumers and health food enterprises to ESS (0,0) and ESS (1,1) under different costs of information acquisition. From the figure, when the strategy of health food enterprises is stable to produce low-quality health food, a lower cost for consumers to search for risk information of health food safety implies faster stability of the strategy. When the strategy of health food enterprises is stable to produce low-quality health food, a lower cost for consumers to search for risk information of health food safety indicates that purchasers' strategy becomes stable faster than nonpurchasers. Therefore, the relationship between health food safety risk and the cost of consumer information acquisition is revealed to a certain extent; that is, a higher information acquisition cost often leads to higher health food safety risks. On the contrary, low cost of health food safety risk information acquisition has a certain effect on containment of health food safety risks.

Figure 2(b) shows that when the strategy of health food enterprises is stable to produce high-quality health food, a lower cost of searching for health food safety risk information implies a faster strategy in stabilizing purchases. Conversely, when the strategy of health food enterprises is stable to produce high-quality health food, a higher cost of consumer search for health food safety risk information indicates a slower strategy in stabilizing purchases.

**Figure 3:**
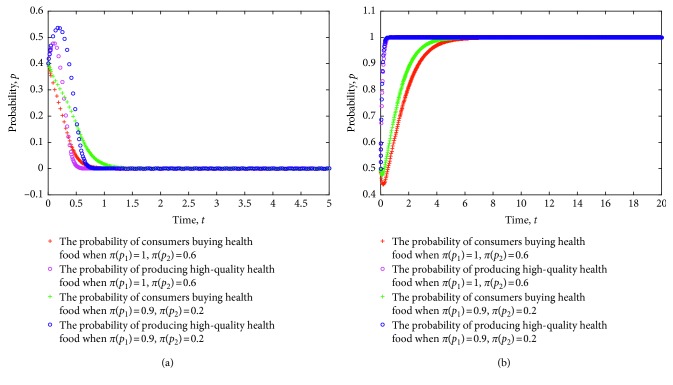
Evolution of strategies of consumers and health food enterprises under different degrees of subjective perception.

The game model is simulated considering the different degrees of accuracy of consumers' subjective perception of health food. The evolution of the strategy of consumers and health food enterprises is shown in Figure 3, where the value of each parameter is set as follows: *e*=8, *L*=5, *P*_h_=9, *P*_l_=7, *R*=3, *a*=30, *b*=0.3, *C*_h_=5, *C*_l_=3.5, *δ*=1.2, *λ*=0.8, and *c*=1.

Figure 3 shows the evolution of strategies for consumers and health food enterprises to ESS (0,0) and ESS (1,1) under different degrees of subjective perception. From the figure, when the strategy of health food enterprises is stable in the production of low-quality health food, a higher accuracy of consumers' subjective perception of health food indicates a faster strategy in stabilizing nonpurchases. Conversely, when the strategy of health food enterprises is stable in the production of low-quality health food, a lower subjective perception of the efficacy of health food implies slower strategy in stabilizing nonpurchases. Particularly, the lower the accuracy of consumers' subjective perception of health food, the more likely they will lead to health food safety risks. When the strategy of health food enterprises is stable to produce high-quality health food, a higher accuracy of consumers' subjective perception of health food indicates a faster strategy in stabilizing purchases. Conversely, when the strategy of health food enterprises is stable to produce high-quality health food, a lower accuracy of consumers' subjective perception of health food indicates a slower strategy in stabilizing purchases.

**Inference 1 .**

Under the constraint conditions of Case 1, that is, (*a* − *bP*_h_)*C*_h_ − (*a* − *bP*_l_)*C*_l_ > 0 and *δ*(*a* − *bP*_h_ − *C*_h_)*P*_h_ − *λ*(*a* − *bP*_l_ − *C*_l_)*P*_l_ > 0, a stronger certification effect of the relevant information of health food enterprises issued by the government aids more in curbing health food safety risks; a higher cost of information search for consumers indicates higher health food safety risks, and a lower accuracy of consumers' subjective perception implies higher health food safety risks.

**Case 2 .**

(*a* − *bP*_h_)*C*_h_ − (*a* − *bP*_l_)*C*_l_ > 0  and  *δ*(*a* − *bP*_h_ − *C*_h_)*P*_h_ − *λ*(*a* − *bP*_l_ − *C*_l_)*P*_l_ < 0.

The constraint conditions of Case 2 indicate that (1) when consumers opt to buy health food, enterprises that produce high-quality health food gain increased profit and (2) when consumers opt not to buy health food, enterprises that produce low-quality health food gain increased profit. These constraint conditions imply that regardless of the strategy of the consumers, the enterprises that produce low-quality health food are increasingly profitable than those that produce high-quality health food.

Table 4 presents the results of local stability analysis under Case 2 based on the values of the elements of the Jacobian matrix in Table 2.

The following conclusions can be drawn from the results of the stability analysis shown in Table 4.

**Theorem 2 .**

Under the constraint conditions (*a* − *bP*_h_)*C*_h_ − (*a* − *bP*_l_)*C*_l_ > 0 and *δ*(*a* − *bP*_h_ − *C*_h_)*P*_h_ − *λ*(*a* − *bP*_l_ − *C*_l_)*P*_l_ < 0, the stable strategy is ESS (0,0).

**Figure 4:**
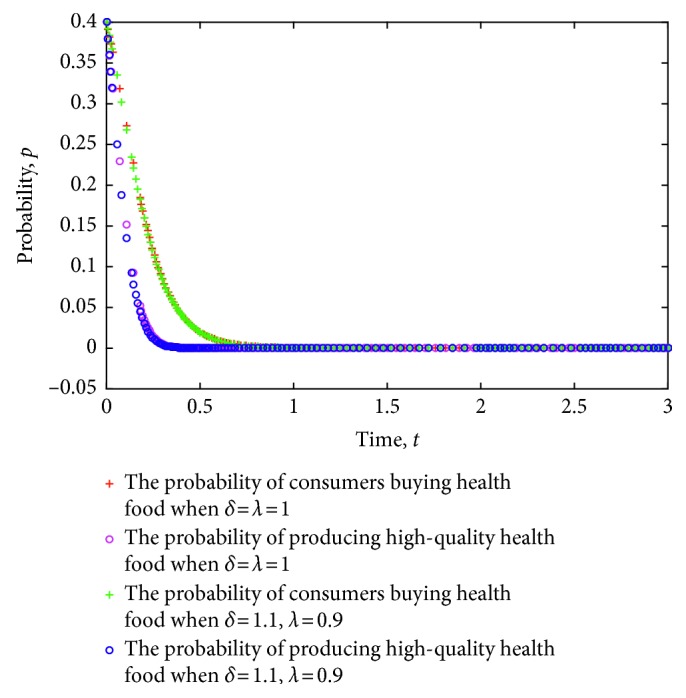
Evolution of strategies of consumers and health food enterprises under different certification effects.

First, the game strategy of consumers and health food enterprises is simulated considering the different certification effects of relevant information on health food enterprises issued by the government, and the evolution of these strategies is obtained.  Figure 4 shows the simulation results, where the value of each parameter is set as follows: *e*=8, *c*=1, *π*(*p*_1_)=0.9, *π*(*p*_2_)=0.6, *L*=5, *P*_h_=9, *P*_l_=7, *R*=3, *a*=20, *b*=1.1, *C*_h_=5, and *C*_l_=4.

As shown in Figure 4, if the relevant information of the government's health food enterprises has a certification effect in the health food market, then the stability of the health food enterprises will be affected. Moreover, the more significant the certification effect, the slower the strategy of health food enterprises in stabilizing the production of low-quality health food. However, the weaker the relevant information of the health food enterprises as issued by the government, the faster the strategy of health food enterprises in stabilizing the production of low-quality health food and the more likely it will lead to health food safety risks.

**Inference 2 .**

Under the constraint conditions of Case 2, that is, (*a* − *bP*_h_)*C*_h_ − (*a* − *bP*_l_)*C*_l_ > 0 and *δ*(*a* − *bP*_h_ − *C*_h_)*P*_h_ − *λ*(*a* − *bP*_l_ − *C*_l_)*P*_l_ < 0, a weaker certification effect indicates higher health food safety risks.

**Figure 5:**
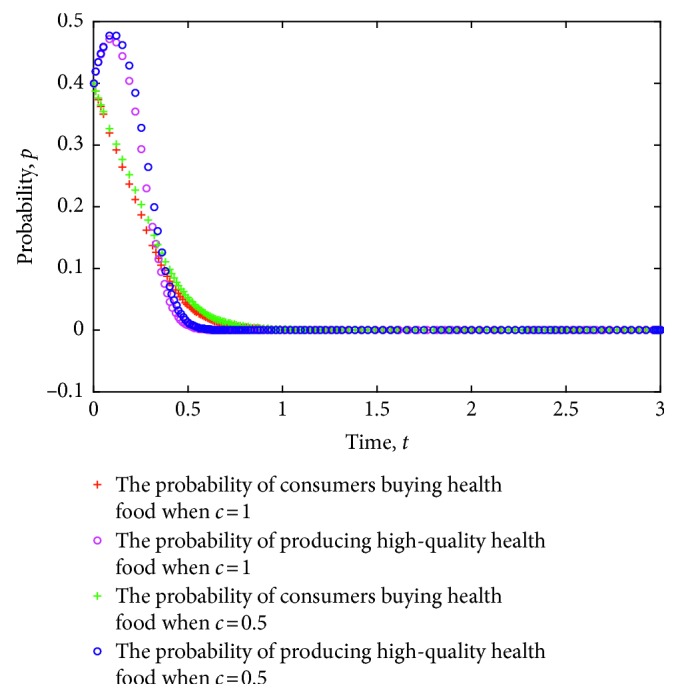
Evolution of strategies of consumers and health food enterprises under different costs of information acquisition.

At the same time, a simulation experiment on the strategy evolution of consumers and health food enterprises is conducted considering the costs of information acquisition.  Figure 5 shows the simulation results, where the value of each parameter is set as follows: *e*=8, *π*(*p*_1_)=0.9, *π*(*p*_2_)=0.6, *L*=5, *P*_h_=9, *P*_l_=7, *R*=3, *a*=20, *b*=1.1, *C*_h_=5, *C*_l_=3.5, *δ*=1, and *λ*=1.

As shown in Figure 5, when enterprises that produce low-quality health food are considerably higher than the profitability of the enterprises that produce high-quality health food, the strategy of health food enterprises will eventually stabilize in the production of low-quality health food. At the same time, a lower cost of information acquisition implies that consumers will faster stabilize a nonpurchasing strategy. By contrast, a higher cost of information acquisition implies that consumers will stabilize a nonpurchasing strategy more slowly. As the strategy of health food enterprises will be stable in the production of low-quality health food, a slower nonpurchasing strategy of consumers indicates higher health food safety risks. Therefore, a high cost of information acquisition frequently leads to health food safety risks. By contrast, a low cost of information acquisition will effectively prevent health food safety risks.

**Inference 3 .**

Under the constraint conditions of Case 2, that is, (*a* − *bP*_h_)*C*_h_ − (*a* − *bP*_l_)*C*_l_ > 0 and *δ*(*a* − *bP*_h_ − *C*_h_)*P*_h_ − *λ*(*a* − *bP*_l_ − *C*_l_)*P*_l_ < 0, when the cost of information acquisition is high, a slower nonpurchasing strategy of consumers of health food implies higher health food safety risks.

**Figure 6:**
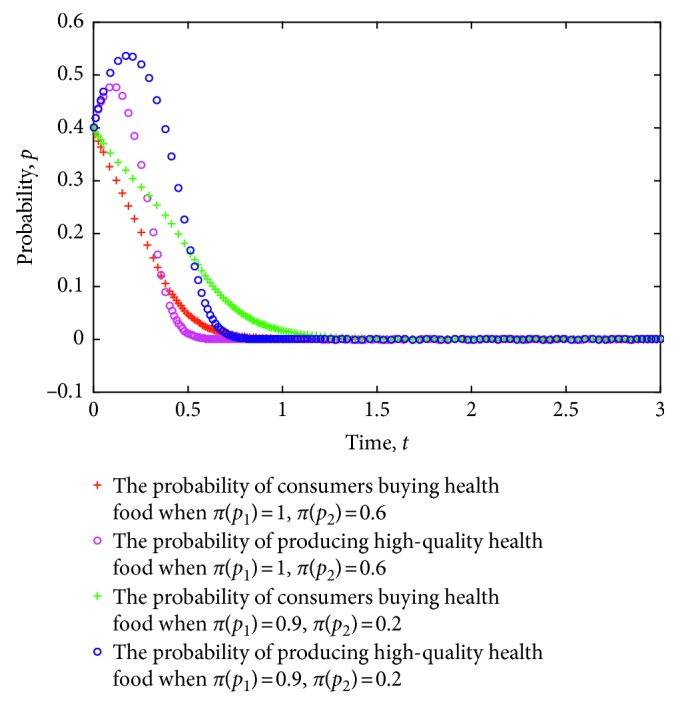
Evolution of strategies of consumers and health food enterprises under different degrees of subjective perception.

The game model is simulated considering the different degrees of accuracy of consumers' subjective perception of health food. The evolution of the strategy of consumers and health food enterprises is shown in Figure 6, where the value of each parameter is set as follows: *e*=8, *c*=1, *L*=5, *P*_h_=9, *P*_l_=7, *R*=3, *a*=30, *b*=0.3, *C*_h_=5, *C*_l_=3.5, *δ*=1.2, and *λ*=0.8.

As shown in Figure 6, the strategy of health food enterprises will eventually stabilize the production of low-quality health food. Meanwhile, health food consumers will gradually stabilize a nonpurchasing strategy. During evolution, a higher accuracy of consumers' subjective perception of health food indicates faster stability in their nonpurchasing strategies. By contrast, a lower accuracy of consumers' subjective perception of health food implies slower stability in their nonpurchasing strategies. Specifically, a higher accuracy of consumers' subjective perception of health food curbs health food safety risks. By contrast, a lower accuracy of consumers' subjective perception of health food indicates higher health food safety risks, thereby endangering the consumers.

**Inference 4 .**

Under the constraint conditions of Case 2, that is, (*a* − *bP*_h_)*C*_h_ − (*a* − *bP*_l_)*C*_l_ > 0 and *δ*(*a* − *bP*_h_ − *C*_h_)*P*_h_ − *λ*(*a* − *bP*_l_ − *C*_l_)*P*_l_ < 0, a lower accuracy of consumers' subjective perception of health implies slower stability of consumers' nonpurchasing strategies, which will more likely cause health food safety risks.

**Case 3 .**

(*a* − *bP*_h_)*C*_h_ − (*a* − *bP*_l_)*C*_l_ < 0  and  *δ*(*a* − *bP*_h_ − *C*_h_)*P*_h_ − *λ*(*a* − *bP*_l_ − *C*_l_)*P*_l_ > 0.

The constraint conditions of Case 3 imply that (1) when consumers opt to buy health food, food enterprises that produce high-quality health food gain increased profit and (2) when consumers do not buy health food, companies that produce high-quality health food lose less. These constraint conditions indicate that regardless of the consumers' strategy on health food and the existence of certification effect, enterprises that produce high-quality health food are considerably better than those that produce low-quality health food.

Table 5 shows the results of local stability analysis under Case 3 based on the values of the elements of the Jacobian matrix (Table 2).

The following conclusions can be drawn from the results of the stability analysis shown in Table 5.

**Theorem 3 .**

Under the constraint conditions (*a* − *bP*_h_)*C*_h_ − (*a* − *bP*_l_)*C*_l_ < 0 and *δ*(*a* − *bP*_h_ − *C*_h_)*P*_h_ − *λ*(*a* − *bP*_l_ − *C*_l_)*P*_l_ > 0, the stable strategy is ESS (1,1).

**Figure 7:**
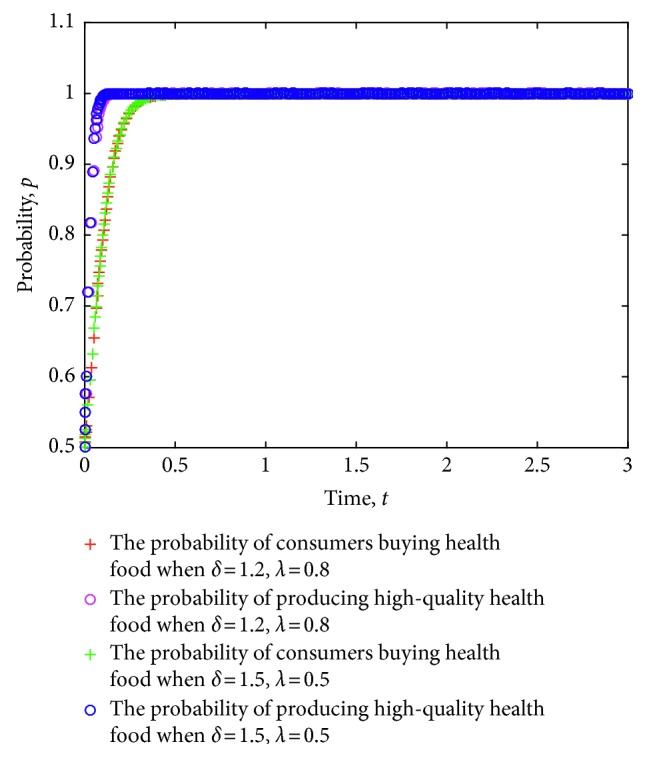
Evolution of strategies of consumers and health food enterprises under different certification effects.

The game strategy of consumers and health food enterprises is simulated considering the different certification effects of the relevant information on health food enterprises as issued by the government. The evolution of the strategies of consumers and health food enterprises is obtained.  Figure 7 shows the simulation results, where the value of each parameter is set as follows: *e*=8, *c*=1, *π*(*p*_1_)=0.9, *π*(*p*_2_)=0.6, *L*=5, *P*_h_=9, *P*_l_=7, *R*=3, *a*=20, *b*=2, *C*_h_=5, and *C*_l_=4.

As shown in Figure 7, the enterprises that produce high-quality health food are more profitable than those who produce low-quality health food in the health food market. The government's certification effect on the information of health food enterprises exerts a certain influence on the strategy of health food enterprises. Specifically, a stronger certification effect has more effective containment of health food safety risks. By contrast, a weaker certification effect generates higher health food safety risks.

**Inference 5 .**

Under the constraint conditions of Case 3, that is, (*a* − *bP*_h_)*C*_h_ − (*a* − *bP*_l_)*C*_l_ < 0 and *δ*(*a* − *bP*_h_ − *C*_h_)*P*_h_ − *λ*(*a* − *bP*_l_ − *C*_l_)*P*_l_ > 0, a weaker certification effect indicates that the health food enterprises will more slowly stabilize the strategy of producing high-quality health food, which will result in higher health food safety risks.

**Figure 8:**
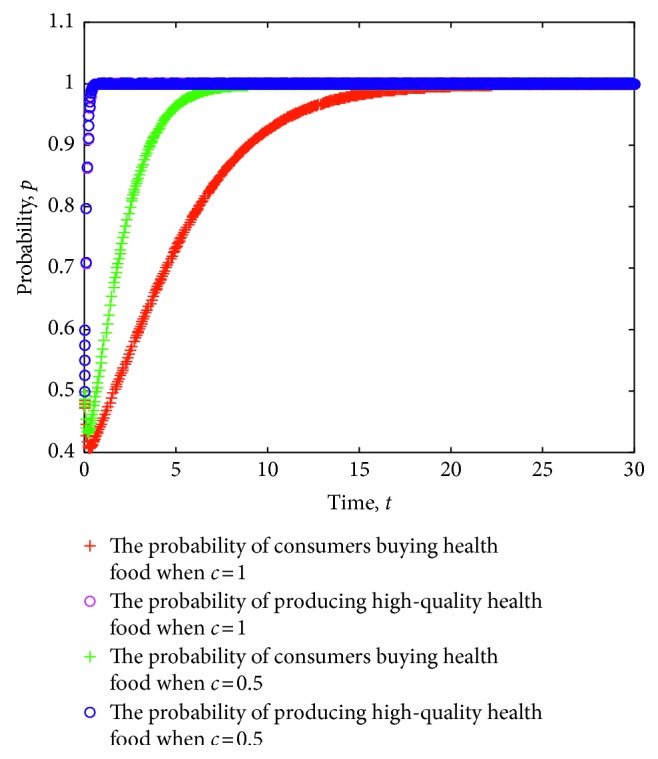
Evolution of strategies of consumers and health food enterprises under different costs of information acquisition.

At the same time, the simulation experiment on the strategy evolution of consumers and health food enterprises is conducted considering the different costs of information acquisition.  Figure 8 shows the simulation results, where the value of each parameter is set as follows: *e*=8, *π*(*p*_1_)=0.9, *π*(*p*_2_)=0.6, *L*=5, *P*_h_=9, *P*_l_=7, *R*=3, *a*=20, *b*=1.1, *C*_h_=5, *C*_l_=3.5, *δ*=1, and *λ*=1.

As shown in Figure 8, when the profitability of the enterprises that produce high-quality health food is obviously higher than those that produce low-quality health food, health food enterprises will eventually stabilize the strategy of producing high-quality health food. At the same time, a lower cost of obtaining information implies a faster stability of the purchase strategy, which will aid in improving the profitability of the enterprises that produce high-quality health food. Conversely, a higher cost of information acquisition indicates a slower stability of the purchase strategy.

**Inference 6 .**

Under the constraint conditions of Case 3, that is, (*a* − *bP*_h_)*C*_h_ − (*a* − *bP*_l_)*C*_l_ < 0 and *δ*(*a* − *bP*_h_ − *C*_h_)*P*_h_ − *λ*(*a* − *bP*_l_ − *C*_l_)*P*_l_ > 0, a higher cost of information acquisition implies a slower stability of the purchase strategy of health food.

**Figure 9:**
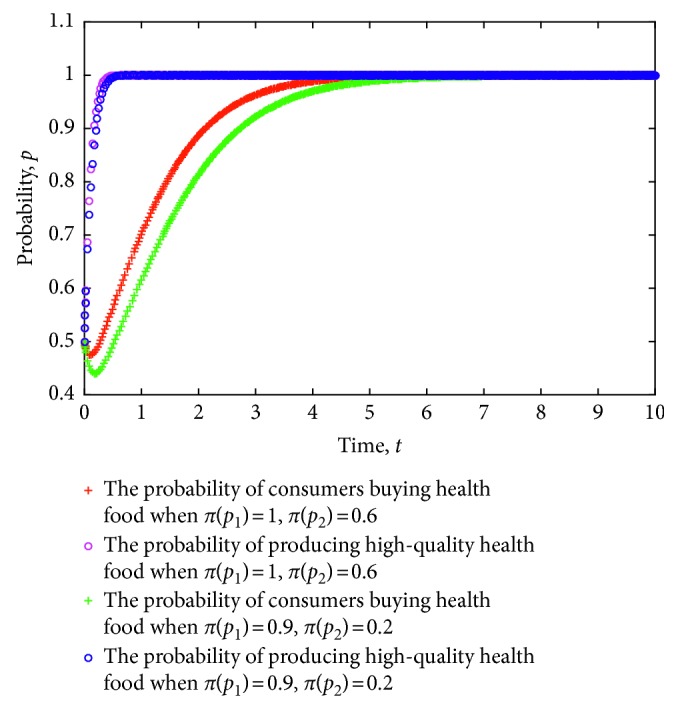
Evolution of strategies of consumers and health food enterprises under different degrees of subjective perception.

The game model is simulated considering the different degrees of accuracy of consumers' subjective perception of health food, and evolution of the strategy of consumers and health food enterprises is depicted.  Figure 9 shows the simulation results, where the value of each parameter is set as follows: *e*=8, *c*=1, *L*=5, *P*_h_=9, *P*_l_=7, *R*=3, *a*=30, *b*=0.3, *C*_h_=5, *C*_l_=3.5, *δ*=1.2, and *λ*=0.8.

As shown in Figure 9, the strategy of health food enterprises will ultimately stabilize the production of quality health food, whereas health food consumers will gradually stabilize the purchase strategy. During evolution, a higher accuracy of consumers' subjective perception of health food implies faster stability of the purchase strategy, which aids in improving the profitability of the enterprises that produce high-quality health food. By contrast, a lower accuracy of consumers' subjective perception indicates slower stability of purchase strategies.

**Inference 7 .**

Under the constraint conditions of Case 4, that is, (*a* − *bP*_h_)*C*_h_ − (*a* − *bP*_l_)*C*_l_ < 0 and *δ*(*a* − *bP*_h_ − *C*_h_)*P*_h_ − *λ*(*a* − *bP*_l_ − *C*_l_)*P*_l_ > 0, a lower accuracy of consumers' subjective perception of health food consumers implies slower stability of purchase strategies.

***Case 4*.**

(*a* − *bP*_h_)*C*_h_ − (*a* − *bP*_l_)*C*_l_ < 0  and  *δ*(*a* − *bP*_h_ − *C*_h_)*P*_h_ − *λ*(*a* − *bP*_l_ − *C*_l_)*P*_l_ < 0.

The constraint conditions of Case 4 indicate that (1) when consumers opt to buy health food, enterprises that produce low-quality health food gain increased profit and (2) when consumers do not buy health food, companies that produce high-quality health food lose less.

Table 6 shows the results of the local stability analysis under Case 4 based on the values of the elements of the Jacobian matrix (Table 2).

The following conclusions can be drawn from the results of the stability analysis shown in Table 6.

**Theorem 4 .**

Under the constraint conditions (*a* − *bP*_h_)*C*_h_ − (*a* − *bP*_l_)*C*_l_ < 0 and *δ*(*a* − *bP*_h_ − *C*_h_)*P*_h_ − *λ*(*a* − *bP*_l_ − *C*_l_)*P*_l_ < 0, the system does not have a stable evolutionary strategy.

**Figure 10:**
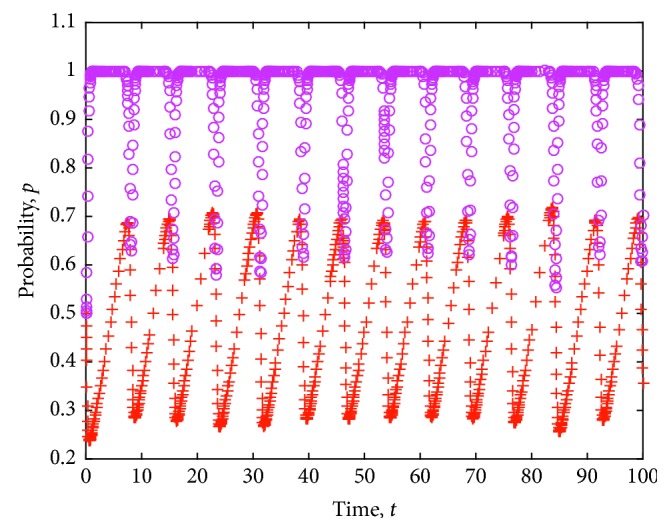
Evolution of strategies of consumers and health food enterprises under different certification effects.

The game strategy of consumers and health food enterprises is simulated considering the different certification effects of the relevant information on health food enterprises as issued by the government. The evolution of the strategies of consumers and health food enterprises is obtained.  Figure 10 shows the simulation results, where the value of each parameter is set as follows: *e*=8, *c*=1, *π*(*p*_1_)=0.9, *π*(*p*_2_)=0.6, *L*=5, *P*_h_=9, *P*_l_=7, *R*=3, *a*=20, *b*=2, *C*_h_=5, and *C*_l_=4.

## 4. Conclusions

We construct a strategic game model, which includes parties, such as government regulators, health food consumers, and health food enterprises, based on prospect theory and depict the evolution of health food enterprises and health food consumers. Factors, such as determining the consumers' ability to identify health food safety information, subjective perception of the efficacy of health food, and the certification effect of regulatory information in the government supervision department, are also considered. Then, the certification effect of information that is issued by the government supervision department, the information search cost of health food consumers, and the subjective perception of health food consumers on the efficacy and safety risk of health food are visualized and analyzed by using computer simulation technology. The following conclusions can be drawn through theoretical and simulation analyses:

1. Under constraint conditions (*a* − *bP*_h_)*C*_h_ − (*a* − *bP*_l_)*C*_l_ > 0 and *δ*(*a* − *bP*_h_ − *C*_h_)*P*_h_ − *λ*(*a* − *bP*_l_ − *C*_l_)*P*_l_ > 0, the stable strategies are ESS (0,0) and ESS (1,1).
2. Under constraint conditions (*a* − *bP*_h_)*C*_h_ − (*a* − *bP*_l_)*C*_l_ > 0 and *δ*(*a* − *bP*_h_ − *C*_h_)*P*_h_ − *λ*(*a* − *bP*_l_ − *C*_l_)*P*_l_ < 0, the stable strategy is ESS (0,0).
3. Under constraint conditions (*a* − *bP*_h_)*C*_h_ − (*a* − *bP*_l_)*C*_l_ < 0 and *δ*(*a* − *bP*_h_ − *C*_h_)*P*_h_ − *λ*(*a* − *bP*_l_ − *C*_l_)*P*_l_ > 0, the stable strategy is ESS (1,1).
4. Under constraint conditions (*a* − *bP*_h_)*C*_h_ − (*a* − *bP*_l_)*C*_l_ < 0 and *δ*(*a* − *bP*_h_ − *C*_h_)*P*_h_ − *λ*(*a* − *bP*_l_ − *C*_l_)*P*_l_ < 0, the system does not have an evolutionary stable strategy.
5. A strong certification effect of the relevant information of the health food enterprises that is issued by the government aids in curbing the risk of food safety. A higher cost of information acquisition for consumers indicates a higher likelihood of health food safety accidents. Meanwhile, lower accuracy of the subjective perception of consumers implies a more possible occurrence of the risk of health food safety.

## Figures and Tables

**Table 1:** Payment matrix.

		Health food enterprises	
		High quality (*y*)	Low quality (1 − *y*)
Consumers	Consumption (*x*)	*V*_1_(*p*_1_, *e*, *c*) − *P*_h_, *δ*(*a* − *bP*_h_ − *C*_h_)*P*_h_	*V*_2_(*p*_2_, *L*, *c*) − *P*_l_, *λ*(*a* − *bP*_l_ − *C*_l_)*P*_l_
	Nonconsumption (1 − *x*)	−*R*, −(*a* − *bP*_h_)*C*_h_	−*R*, −(*a* − *bP*_l_)*C*_l_

**Table 2:** Value of elements in the Jacobian matrix.

	*a*_11_	*a*_12_	*a*_21_	*a*_22_
(0,0)	*βπ*(*p*_2_)(−*L* − *c*) − *P*_l_+*R*	0	0	−(*a* − *bP*_h_)*C*_h_+(*a* − *bP*_l_)*C*_l_
(0,1)	*απ*(*p*_1_)(*e* − *c*) − *P*_h_+*R*	0	0	(*a* − *bP*_h_)*C*_h_ − (*a* − *bP*_l_)*C*_l_
(1,0)	−*βπ*(*p*_2_)(−*L* − *c*)+*P*_l_ − *R*	0	0	*δ*(*a* − *bP*_h_ − *C*_h_)*P*_h_ − *λ*(*a* − *bP*_l_ − *C*_l_)*P*_l_
(1,1)	−*απ*(*p*_1_)(*e* − *c*)+*P*_h_ − *R*	0	0	−*δ*(*a* − *bP*_h_ − *C*_h_)*P*_h_+*λ*(*a* − *bP*_l_ − *C*_l_)*P*_l_
(*x*^*∗*^, *y*^*∗*^)	0	Λ	Γ	0

**Table 3:** Results of local stability analysis under Case 1.

	Symbols of each element in the Jacobian matrix				*det J*	*tr J*	Stability
	*a*_11_	*a*_12_	*a*_21_	*a*_22_			
(0,0)	−	0	0	−	+	−	ESS
(0,1)	+	0	0	+	+	+	Unstable
(1,0)	+	0	0	+	+	+	Unstable
(1,1)	−	0	0	−	+	−	ESS

**Table 4:** Results of local stability analysis under Case 2.

	Symbols of each element in the Jacobian matrix				*det J*	*tr J*	Stability
	*a*_11_	*a*_12_	*a*_21_	*a*_22_			
(0,0)	−	0	0	−	−	+	ESS
(0,1)	+	0	0	+	+	+	Unstable
(1,0)	+	0	0	−	−	−	Unstable
(1,1)	−	0	0	+	+	−	Unstable

**Table 5:** Results of local stability analysis under Case 3.

	Symbols of each element in the Jacobian matrix				*det J*	*tr J*	Stability
	*a*_11_	*a*_12_	*a*_21_	*a*_22_			
(0,0)	−	0	0	+	−	Uncertain	Unstable
(0,1)	+	0	0	−	−	Uncertain	Unstable
(1,0)	+	0	0	+	+	+	Unstable
(1,1)	−	0	0	−	+	−	ESS

**Table 6:** Results of local stability analysis under Case 4.

	Symbols of each element in the Jacobian matrix				*det J*	*tr J*	Stability
	*a*_11_	*a*_12_	*a*_21_	*a*_22_			
(0,0)	−	0	0	+	−	Uncertain	Unstable
(0,1)	+	0	0	−	−	Uncertain	Unstable
(1,0)	+	0	0	−	−	Uncertain	Unstable
(1,1)	−	0	0	+	−	Uncertain	Unstable

## Data Availability

The method in this article is computer mathematical simulation. Numerical simulation analysis is the most effective way to test real-time dynamic data without a large number of empirical validations. We simulate the influence mechanism of these factors, including the cost of searching for health food information, consumers' subjective perception of health food efficiency, and the certification effect of supervision departments, on health food safety risk evolution by using Matlab2016b software. Thus, this paper does not have the data that can be obtained because we directly use the plot function of Matlab2016b software to make the images.
